# Burden of human metapneumovirus infections among children with acute respiratory tract infections attending a Tertiary Care Hospital, Kathmandu

**DOI:** 10.1186/s12887-023-04208-6

**Published:** 2023-08-07

**Authors:** Jyoti Lamichhane, Milan Upreti, Krishus Nepal, Bishnu Prasad Upadhyay, Urusha Maharjan, Ram Krishna Shrestha, Ram Hari Chapagain, Megha Raj Banjara, Upendra Thapa Shrestha

**Affiliations:** 1 GoldenGate International College, Battisputali, Kathmandu Nepal; 2 Central Diagnostic Laboratory & Research Center, Kamalpokhari, Kathmandu Nepal; 3 Kanti Children’s Hospital, Maharajgunj, Kathmandu Nepal; 4 Central Department of Microbiology, Tribhuvan University, Kirtipur, Kathmandu Nepal

**Keywords:** Respiratory tract infections, Human metapneumovirus, Multiplex real-time RT-PCR, Pneumonia, Bronchiolitis

## Abstract

**Background:**

Acute respiratory infections (ARIs) are one of the most common causes of mortality and morbidity worldwide. Every year millions of children suffer from viral respiratory tract infections (RTIs) ranging from mild to severe illnesses. Human Metapneumovirus (HMPV) is among the most frequent viruses responsible for RTIs. However, HMPV infections and their severity among children have not been explored yet in Nepal.

**Purpose:**

Therefore, the study was focused on HMPV infections and other potential viral etiologies or co-infections using multiplex PCR among children attending Kanti Children’s Hospital and assessed the clinical characteristics of the infections as well as found the co-infections. A hospital-based cross-sectional study was designed and a convenience sampling method was used to enroll children of less than 15 years with flu-like symptoms from both outpatients and inpatients departments over three months of the study period.

**Results:**

HMPV infection (13.3%) was the most predominant infection among the different viral infections in children with ARIs in Kanti Children’s Hospital. The HMPV was more prevalent in the age group less than three years (21.8%). Cough and fever were the most common clinical features present in all children infected with HMPV followed by rhinorrhea, sore throat, and wheezing. HMPV-positive children were diagnosed with pneumonia (42.9%), bronchiolitis (28.5%), upper respiratory tract infections (14.3%), and asthma (14.3%). The prevalence of HMPV was high in late winter (14.3%) followed by early spring (13.5%).

**Conclusions:**

This study provides the baseline information on HMPV and associated co-infection with other respiratory viruses for the differential diagnosis based on molecular methods and also the comparison of clinical presentations among the different respiratory syndromes.

## Introduction

Acute respiratory infections (ARIs) pose a major public health problem worldwide with significant morbidity and mortality [1, 2]. ARIs can be classified into upper respiratory tract infections (URIs) and lower respiratory tract infections [3]. Globally, 17.2 billion URIs alone have been reported in 2019 [4]. About 13 million children less than 5 years of age die every year. About 95% of them are from developing countries and one-third of the total deaths are due to ARIs [5]. The incidence of severe ARIs is the highest in Southeast Asian and African regions [6]. Bangladesh, India, Indonesia, and Nepal together report a total of 40% global mortality due to ARIs. It is estimated that 30–50% of hospital visits and 20–40% of hospital admission is related to ARIs [7]. Likewise, 68.06 million episodes of lower respiratory infections have been reported in 2016 [6]. Pneumonia and bronchiolitis are the leading contributors to the global burden of ARIs in young children. These infections are responsible for the greater part of the mortality rate, of which the vast majority occur in developing countries. Pneumonia alone killed 740,180 children under the age of 5 in 2019 which accounts for 14% of all deaths [8].

Viral agents are among the most common pathogens responsible for respiratory tract infections in young children [9, 10] and also in adults [11–13]. However, the etiological agents for a large number of respiratory tract infections (RTIs) remain unknown [14]. Respiratory syncytial virus (RSV), Parainfluenza virus (PIV), Influenza virus, and Adenovirus have been considered the leading causes of acute viral respiratory tract infections [15, 16]. A study in Thailand from Jan 2015 to Dec 2019 reported RSV as the main etiology of bronchiolitis followed by Influenza viruses were the most predominant among children with pneumonia (15.52%) [17]. Since the advent of more sensitive diagnostic tools, like PCR, the proportion of known viral etiologies has increased. In the last ten years due to the advancement in molecular technologies, newly discovered viruses have been identified from patients with RTIs, like Human Metapneumoviruses, Coronaviruses NL63, and HKU, Human Bocaviruses, new Enteroviruses, Parechoviruses, Rhinovirus strains, Polyomaviruses WU and KI and the pandemic H1N1 influenza A virus [15].

HMPV can infect people of all age groups, with a high prevalence in pediatric patients, older adults, and immunocompromised individuals [18, 19]. Infections with HMPV can be both symptomatic and asymptomatic [20]. Clinical manifestations of symptomatic HMPV are indistinguishable from those of RSV and range from mild upper respiratory tract infection to severe diseases requiring hospitalization like severe cough, bronchiolitis, and pneumonia, often accompanied by high-grade fever, myalgia, and vomiting [21]. The incubation period is estimated to be 3 to 6 days, and the median duration of illness can vary depending upon severity but is similar to other respiratory infections caused by other viruses [18]. Re-infection with HMPV is common [20]. To date, there is no vaccine is available [18].

HMPV infections can occur throughout the year, but seasonality has been described in several studies [22, 23]. The seasonal distribution of HMPV was found to be largely similar to that of RSV, with the peak of virus detection in winter [11]. However, it varies from year to year and from place to place [15].

Since the advancement of molecular techniques, the detection of co-infections by multiple respiratory viruses from the same respiratory specimen has been made possible. In children hospitalized due to severe bronchiolitis, co-infection may reach 70% according to some reports, although most studies have shown that prevalence rates range from 15 to 39% [24, 25]. Because Human metapneumovirus is relatively new and not well described, the chances of underdiagnosis in regular medical practice are very high. Center for Disease Control and Prevention (CDC) recommends considering HMPV testing along with Influenza virus (Flu), RSV, and other common respiratory viruses, especially in patients with severe respiratory illness during winter and spring, when HMPV is highly circulating [26]. Despite that, there are only limited reports regarding HMPV infections. Studies had shown that HMPV is one of the prevalent causes of respiratory tract infections in South Asian countries including Nepal [27, 28]. Dr. Mathisen (2010) found that about 4.2% of pneumonia in children in Bhaktapur, Nepal was caused by HMPV [27]. Women in Nepal with HMPV during pregnancy had an increased risk of giving birth to infants who were small for gestational age [28].

Actual data regarding the prevalence of HMPV alone in our country is not known however some studies had described the co-infection of HMPV with other viruses [29] and bacteria [30]. In addition to the limited data regarding the epidemiology of HMPV in children in subtropical regions such as Nepal; the seasonal pattern remains unknown. Many studies also lack the correlation of clinical syndromes with HMPV infections. On the other hand, using multiplex real-time PCR for diagnosis is rarely used in our context, the study aimed to focus on the diagnosis of HMPV infections and other potential viral etiologies or co-infections among children using multiplex real-time RT-PCR visiting a tertiary care hospital in Kathmandu, Nepal.

## Materials and methods

### Study approval and consent

The study was approved by the review committee, Kanti Children’s Hospital, Maharajgunj, Kathmandu, Nepal (Reference no.: 2,018,019). The study was carried out according to the principles stated in the Declaration of Helsinki (Ethical principles for medical research involving human subjects). Before obtaining the consent, detailed information about the research including study aims, description of the sample collection procedure, potential benefits and risks, and assurance of confidentiality for all information and results were provided to each participant. All participants were also informed about their right to withdraw consent at any time without providing any reason for withdrawal or having fear of any negative consequences. Written informed consent was taken from the legal representatives, either parents or guardians of children. Assent was taken from the participants ≥ 11 years and < 15 years of children.

### Inclusion and exclusion criteria

#### Study population and timeline

The study was carried out in Kanti Children’s Hospital, a tertiary care hospital in Maharajgunj, Kathmandu. Children below 15 years suspected of influenza-like illness with respiratory tract infection (including both influenza-like illness and severe acute respiratory infection cases) as defined by Fitzner et al. 2018 [31] were enrolled in the study over three months period. Children from both outpatients department and the inpatients department were enrolled, however, patients with specific symptoms other than flu-like illness were excluded.

### Sample size

Based on a review by Panda et al. [32], HMPV is the major etiological agent responsible for about 5–10% of hospitalizations of children suffering from acute respiratory tract infections. Considering an average of 7.5% prevalence of HMPV, the sample size was calculated to be 107 [${n=Z}^{2}\frac{p.q}{e2}$, z = 1.96, p = 0.075, q = 0.925, e = 5%]. A convenience sampling method was used to enroll all the patients that meet the inclusion criteria throughout the study period.

### Data collection

The clinical history of HMPV-infected children was obtained from pediatricians’ notes as the notes had more information than the hospital record. Clinical data including demographic data (sex, age, and underlying disease of the patient), clinical symptoms (cough, rhinitis, body temperature, dyspnea, wheezing, feeding difficulties, retractions, headache), and clinical diagnosis (URTI, pneumonia, bronchiolitis, asthma) were recorded from the notes.

### Sample collection

Throat swabs from enrolled patients were collected using a standard microbiological technique for three fixed days every week as per the availability of one of our co-investigators, a Pediatrician from January 2019 to March 2019. The samples were collected in a clean, dry, and sterile Dracon swab stick, which was then immediately kept in a tube containing viral transportation media (VTM) and transported to Central Diagnostic Laboratory and Research Center, Kamalpokhari, Kathmandu within an hour of collection. Samples were stored at -80^o^C until further processing.

### Extraction and purification of viral nucleic acids

RNA was isolated and purified from a 200 µl cell-free sample (supernatant) by using the PureLink™ Viral RNA/DNA Mini kit (Invitrogen, Thermo Fisher Scientific), the entire procedure was carried out according to kit instructions.

### Polymerase chain reaction

All the throat swab samples were tested for HMPV and co-infection with other potential viruses such as RSV, Influenza, Parainfluenza, and Adenovirus by one-step real-time RT-PCR. The viral RNA is transcribed into cDNA using a specific primer-mediated reverse transcription step followed immediately in the same tube by a polymerase chain reaction. The presence of specific viral sequences in the reaction is detected by an increase in fluorescence observed from the relevant dual-labeled probe and is reported as a cycle threshold value (Ct) by the Real-Time thermocycler. The preparation of PCR was done with a master mix (Fast Tract Diagnostica, Luxemburg, Finland) following the manual, FTD Respiratory Pathogens 21 [33]. Reagents for the reaction: the positive control (PC), and 2x RT-PCR buffer were thawed completely and the reaction mix was prepared as shown in Table 1. The- 96 well reaction was placed. 10 µl of the extracted samples, the extracted negative control, and the positive control were added in wells and labeled correctly. Each run included a negative and positive control. The reaction mix with samples/PC/NC was mixed well by pipetting up and down. The plate was closed with the ABI optical adhesive film and briefly centrifuged afterward. Then, the plate was put in real-time thermal cycler Cfx 96, Bio-Rad, USA.

**Table 1:** The amounts of reagents needed for 1, 15, 32, and 64 wells

Number of reactions		1	15	32	64
FTD-2- 32/64	Buffer	12.5 µl	187.5 µl	400 µl	800 µl
	PPmix	1.5 µl	22.5 µl	48 µl	96 µl
	Enzyme	1 µl	15 µl	32 µl	64 µl
	Total	15 µl	225 µl	480 µl	960 µl

RT-PCR amplification was performed with the following cycling parameters: 42 °C for 15 min hold; 94 °C for 3 min hold; 40 cycles of 94 °C for 8 s and 60 °C for 34 s [33]. The fluorescent dyes selected for the multiplex PCR amplification were FAM (∼520 nm), VIC (∼550 nm), ROX (∼610 nm), and Cy5 (∼670 nm) (Table 2).

**Table 2:** Settings of the detectors for the detection of multiple pathogens

PP mix	Pathogens	Dye	Detection wavelength (nm)
**FluRhino PP** (Probe/Primer mix for FLUA, RV, FLUB, H1N1)	FLUA (Influenza A)	green	520
	RV (Human rhinoviruses)	yellow	550
	FLUB (Influenza B)	orange	610
	H1N1 (Influenza A subtypes))	red	670
**Cor PP** (Probe/Primer mix for Cor299, Cor 63, HKU, Cor 43)	Cor 229 (Human coronavirus-299)	green	520
	Cor 63 (Human coronavirus-63)	yellow	550
	HKU ((Human corona virus-HKU1)	orange	610
	Cor 43 (Human coronavirus-43)	red	670
**ParaEAV PP** (Probe/Primer mix for HPIV2-4, IC)	HPIV3 (Human parainfluenza virus 3)	green	520
	HPIV2 (Human parainfluenza virus 2)	yellow	550
	HPIV4 (Human parainfluenza virus 4)	orange	610
	IC (EAV)- Internal control-Equine arteritis virus)	red	670
**BoMpPf1 PP** (Probe/Primer mix for HPIV1, HMPV A/B, HboV, Mpneu)	HPIV1 (Human parainfluenza virus 1)	green	520
	HMPV A/B (Human Metapneumovirus A & B)	yellow	550
	HBoV (Human Boca virus)	orange	610
	Mpneu (Mycoplasma pneumoniae)	red	670
**RsEPA PP** (Probe/Primer mix for HRV A/B, HPeV, EV, HAdV)	HRSVA/B (Human respiratory syncytial virus A & B)	green	520
	HPeV (Human Parechoviruses)	yellow	550
	EV (Entero virus)	orange	610
	HAdV (Human adenovirus)	red	670

### Quality control

A threshold was set according to manufacturer instructions. All negative controls were below the threshold and positive control showed the positive (i.e. exponential) amplification curve. Internal control also showed the positive (i.e. exponential) amplification tract as well. Therefore, the process is valid. After all, the controls met the specified ranges, and all samples were checked for positive traces. Ct results for all color channels were displayed on the “View Well Table” window.

### Data Analysis

All the data were entered and analyzed by using Statistical Package for Social Science (SPSS) version 24 software package. Chi-square tests were performed to assess the difference in results between different groups (age and sex groups). In addition to Chi-square tests, Fischer’s exact tests were used in the data where the total number was less than 5 in any cell of a 2*2 table - such as while assessing the difference in HMPV prevalence among patient types. The result having a p-value < 0.05 was considered significant.

## Results

### Prevalence of different viral infections among children with ARIs

A total of 105 non-duplicate throat swabs were collected from the participants. Out of 105 specimens collected from children with ARIs, 23 (21.9%) were positive for different viral infections by multiplex-PCR. Out of 23 positive cases, 19 were infected with a single viral infection while 4 were co-infected with multiple viruses. Among different viral infections, HPMV infections (Details of HPMV infection given in Table 3) were found to be the most predominant one followed by RSV infection. Out of 5 RSV infections, 4 were from the age group of fewer than 3 years and 1 from the age group of 3–5 years. Likewise, 1 adenoviral infection was detected in the age group of 3–5 years. Of 2 Influenza infections, 1 from each age group of fewer than 3 years and 3–5 years were reported (Table 4).

**Table 3:** Prevalence of HMPV infections in the study population

Distribution of HMPV		Total no.	No. of HMPV positive cases (%)	No. of HMPV negative cases (%)	p-value
**Gender**	Female	46	6 (13)	40 (87)	0.512
	Male	59	8 (13.6)	51 (86.4)	
**Age Group (yrs)**	< 3	32	7 (21.9)	25 (78.1)	0.115
	3–5	45	6 (13.3)	39 (86.7)	
	6–15	28	1 (3.6)	27 (96.4)	
**Patient Type**	Out-patient	90	12 (13.3)	78 (86.7)	1.000
	In-patient	15	2 (13.3)	13 (86.7)	

**Table 4:** Distribution of different viral infections among patients with acute respiratory infections (n = 105)

S. no.	Different viral infections	Mode of infections		No. of positive cases (%)
		Mono infection (%)	Co-infection (%)	
1	HMPV	12	2	14 (13.3)
2	RSV	4	1	5 (4.8)
3	Adenovirus	1	0	1 (0.9)
4	Influenza	2	0	2 (1.9)
5	Parainfluenza	0	1	1 (0.9)
	**Total cases**	**19**	**4**	**23 (21.9)**

***Note***: HMPV: Human Metapneumovirus; RSV: Respiratory syncytial virus

### Distribution of HMPV infection among different study populations

Out of 105 specimens collected from children with ARIs, 14 (13.3%) were positive for HMPV by multiplex-PCR. The prevalence of HMPV among children was observed to be 13.3% (95% CI: 7.0–20). The positive rate of HMPV in females was 13% and the male was 13.6%. HMPV-infected children were aged from newborn to 14 years (median age: 4 years). Most infected individuals were from the outpatient department (Table 3).

### Clinical characteristics of a patient infected with HMPV

The most common clinical findings of HMPV infection were cough (n = 14; 100%) and fever (n = 14; 100%) followed by rhinorrhea (n = 10, 71.4%), sore throat (n = 9; 64.3%), and wheezing (n = 7; 50%). Three of the patients (n = 3; 21.4%) had complaints of loss of appetite and one (n = 1; 7.1%) had a headache. Out of 14 HMPV-positive cases, 42.9% (n = 6) of the patients were diagnosed with pneumonia. Similarly, 28.6% (n = 4) with bronchiolitis, 14.3% (n = 2) were diagnosed with URTI, and 14.3% (n = 2) with asthma (Table 5).

**Table 5:** Clinical characteristics of HMPV-positive patients

Age group (years)	Sex	Symptoms	Clinical diagnosis
< 3	F	Fever, Cough, loss of appetite, Sore Throat	Pneumonia
< 3	F	Fever, Cough, Rhinorrhea, Wheezing, Sore Throat	Asthma
< 3	M	Fever, Cough, Rhinorrhea, loss of appetite, Sore Throat	Pneumonia
< 3	M	Fever, Cough, Rhinorrhea, Wheezing, Sore Throat	Bronchiolitis
< 3	M	Fever, Cough, Wheezing, Sore Throat	Asthma
< 3	M	Fever, Cough, Rhinorrhea, Sore Throat	Bronchiolitis
< 3	M	Fever, Cough, Rhinorrhea, Wheezing, Sore Throat	Pneumonia
3–5	F	Fever, Cough, Rhinorrhea	URTI
3–5	F	Fever, Cough, Loss of Appetite	Pneumonia
3–5	F	Fever, Cough, Rhinorrhea, Wheezing	Bronchiolitis
3–5	M	Fever, Cough, Rhinorrhea, Sore Throat	Bronchiolitis
3–5	M	Fever, Cough, Rhinorrhea, Wheezing	Pneumonia
3–5	M	Fever, Cough, Rhinorrhea, Wheezing	Pneumonia
6–15	F	Fever, Cough, Sore Throat, Headache	URTI

Note: M: Male, F; Female, URTI: Upper respiratory tract infection

### Seasonal variation of HMPV

**Fig. 1:**
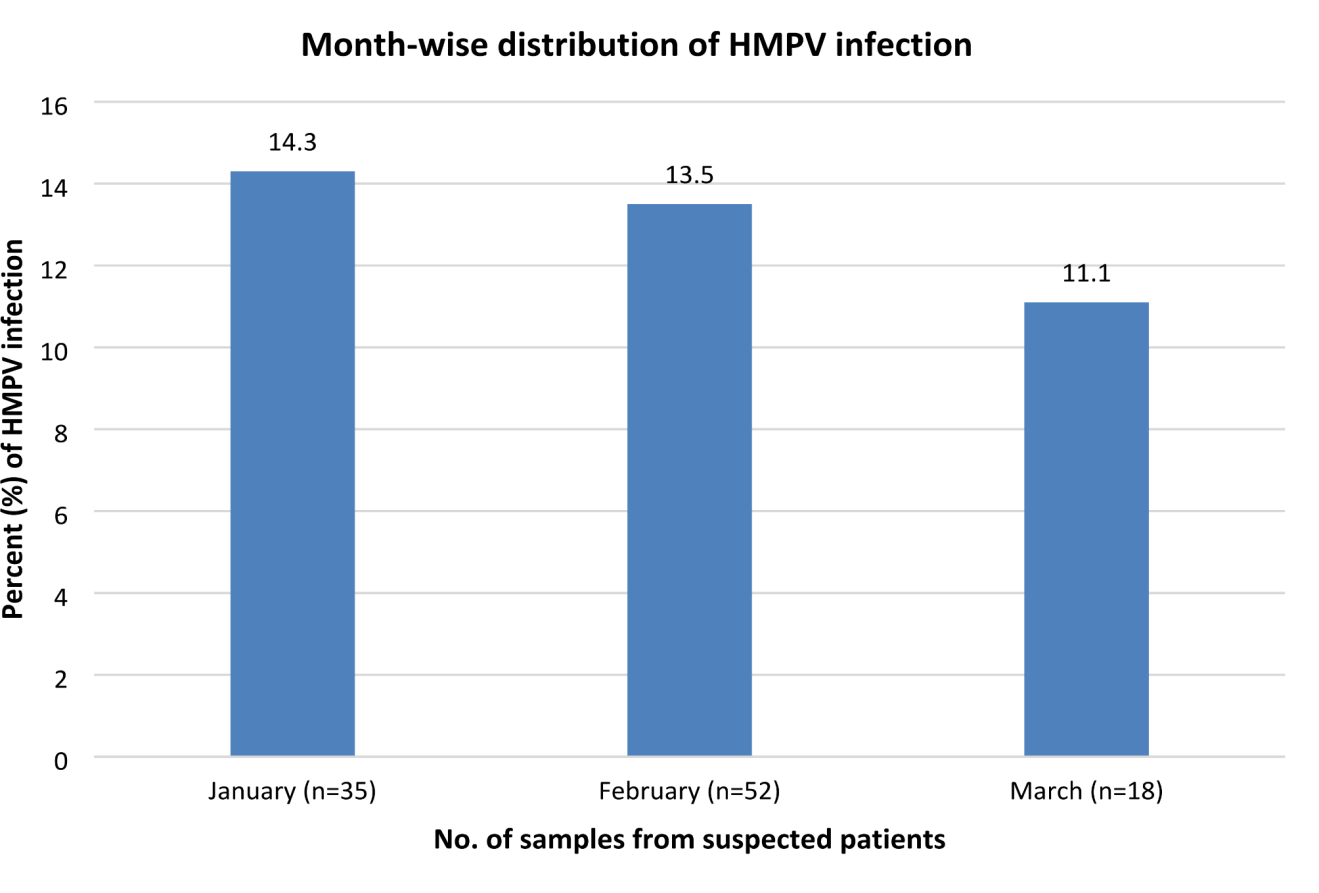
Month-wise distribution of HMPV infection among suspected children

35 samples were collected in January. Similarly, 52 and 18 samples were collected during the second (February) and third months (March) respectively. Out of three months of observation, the rate of HMPV infection was slightly higher in January (14.3%) as compared to February (13.5%) and March (11.1%) which is statistically not significant p > 0.05) (Fig. 1).

### HMPV co-infection with other viruses

Among 14 HMPV-positive samples, co-infection was seen in two different patients. The co-infection with Parainfluenza virus was detected in 7.1% (n = 1) of patients age group < 3 and in 7.1% (n = 1) of the patient with RSV in of age group 3–5 years. No co-infection was observed in patients in the age group 6–15 years. Both co-infection cases were found in children in the outpatient department (Table 6).

**Table 6:** Co-infection of HMPV with other bacteria and viruses among different age groups

Age group	Total positive sample	Co-infection		Total
		RSV (%)	PIV (%)	
< 3	7	0	1 (7.1%)	1
3–5	6	1 (7.1%)	0	1
6–15	1	0	0	0
**Total**	**14**	**1**	**1**	**2**

## Discussion

Since 2001, after the discovery of HMPV, several studies have provided important information on HMPV epidemiology, clinical symptoms associated with HMPV infection, and the patient groups that are at risk for HMPV infection [11, 13, 34–37]. Although few studies have suggested that HMPV is an important causative pathogen in children of Nepal [27, 28], the actual data regarding HMPV infection in the country’s population remains largely unknown due to the limitations of these studies. In this study, we describe the prevalence of HMPV, co-infections with other viruses, and clinical presentation of HMPV, in a Kanti Children’s Hospital setting, and compared the clinical characteristics among those HMPV-positive cases.

Out of 105 specimens collected from children with ARIs, 23 (21.9%) were positive for different viral infections by multiplex-PCR including HMPV. However, studies from the adjoining countries have reported a higher rate of viral infection among children with ARIs. A study from India has reported 82.7% of viral infections in children of less than 5 years with ARIs [9]. Likewise, a study from China, viral infections were diagnosed in 44.9% of children under 5 years with ARIs [10]. Although we use a similar technique for diagnosis, the lower rate of viral infections in our study might be due to only processing of throat swabs. The combined nasopharyngeal and throat swab would increase the detection of more viruses. The use of only supernatant for RNA extraction might have contributed to the lower detection of viruses in this study. This is one of the major study limitations of our research.

HMPV was found in 13.3% of children with ARIs followed by 4.8% of RSV infections and 1.9% of Influenza infections. A review by Shafagati and Williams 2018, has reported the association of HMPV to be 6–40% among acute respiratory illness children [38]. Different studies from different parts of the world have reported variable frequencies of HMPV infection, ranging from 2.2 to 43% [39–43], with young children being the main groups in which HMPV infections are detected. Perchetti et al. 2021 reported a 5.3% prevalence of HMPV infections among infants with respiratory symptoms from rural areas in Nepal followed by RSV infections [44]. A number of studies reported an increased rate of HMPV infection among children under 5 years [44–46]. Since this study was only limited to three months’ duration, it would be rather difficult to conclude the epidemiology of HMPV among Nepalese children. However, during that brief study period, we observed HMPV as the most prevalent virus detected in children with ARIs. Our study showed that HMPV is an important contributor to ARI, and is associated with substantial pneumonia burden in children. In addition, we found a slightly higher rate of HMPV infections among children of age group < 3 years is higher (21.8%) as compared to HMPV infections of 13.3% and 3.5% in age groups 3–5 years and 6–15 years respectively, the data was statistically not significant. Likewise, a survey conducted in Kenya for 10 years among children has reported the highest prevalence among children below 2 years of age group [47]. A more detailed study on the diagnosis of ARIs in children including different specimens for at least one year would be required to make any conclusion on the epidemiology and public health importance of HMPV and its potential severity to children.

A year-round survey that included children aged less than 5 years reported that the burden of HMPV infection is up to 7% in the United States [36]. It has been reported that the higher burden is among children from low-income settings. A study from North East Brazil reported a prevalence of HMPV of 10% in preschool children [48]. Our study suggests that the positive rate in this age group might be at least as high, however further study in this population is required.

No HMPV-positive children aged 6 to 15 years were hospitalized. Rates of HMPV-associated hospitalization are highest among children < 3 years old. A similar result was reported by Edward et al. 2013 [36]. The higher prevalence of HMPV and disease severity in young children is multifactorial and includes elements of immunological naïveté [49], infecting dose exposure and crowding, other siblings, vaccination status against other respiratory pathogens, ventilation, prior respiratory and proximal diarrhoeal disease and its impact on nutritional status, the anatomy of the chest wall and lung physiology in young children, as well as other factors, as noted in other studies [50].

In this study, we found the most common clinical symptoms of HMPV associated with ARTI were fever, cough, rhinorrhea, sore throat, and wheezing. Among the 14 HMPV-positive children, cough, fever, rhinorrhea, and sore throat were the most common features. Wheezing was present in approximately half of the children. No earache or post-tussive emesis was found. Comparable data was reported in a study by Honda et al. [51]. Several reports indicate that HMPV is a commonly identified cause of pediatric lower RTIs, and is second only to RSV as a cause of bronchiolitis in early childhood [11]. A study that included children diagnosed with acute lower RTI reported bronchiolitis as the most common presentation of HMPV illness [52]. However, in our study, pneumonia was the most common clinical diagnosis followed by bronchiolitis and asthma. There were no cases of otitis media. Cong et al. 2022 [53] and Wang et al. 2021 [54] have also reported pneumonia (92.7% and 55.77% respectively) as a main diagnosed disease among HMPV-positive children.

The seasonal distribution of HMPV infection varies [55]. Some longitudinal studies [22, 44, 45] suggested that the high season for HMPV is from winter to spring (between December and May) and the low season is the fall (around September and October). Wang et al. 2021 and Cong et al. 2022, have reported the prevalence of HMPV higher during the winter and autumn seasons [53, 54]. However, the high season for HMPV in tropical and subtropical areas varies from winter to spring in Brazil, spring and/or summer in Taiwan, and the rainy season in Vietnam [22]. This study was a brief duration of observation for only the first three months of the year. Further longitudinal study would be required for us to document the seasonality for this setting. Samples from different areas should be tested.

Due to the similar seasonal distribution of HMPV and other respiratory viruses, the potential co-infection likely existed. Some studies have found a co-infection rate of up to 70% in HMPV [24, 25, 56]. In this study, the majority of HMPV-infected cases did not show any co-infections. Only two out of 14 positive patients were co-infected with other viruses. The co-infected viruses included respiratory syncytial virus and parainfluenza virus. Besides RSV and Parainfluenza virus, a longitudinal study conducted in rural areas in Nepal reported Rhinovirus, Adenovirus, and Bocavirus co-infections with HMPV among infants with respiratory symptoms [44]. The absence of more respiratory viruses in this study might be due to convenience sampling without controls for a brief study duration. In addition, the small sample size can distort the findings of the study from the regular trend, simply by random effects [57]. Nevertheless, the lack of other respiratory pathogens in most patients suggests that HMPV is a major pathogen of both the upper and lower respiratory tracts in this study.

Kanti Children’s Hospital is one of the major children’s hospitals in Nepal and the patients come from all over the country, so the data presented here represent the scenario of the country during the study period. However, as a major study limitation, we could not conduct our study throughout the year including all seasons because of a limited budget and time. Therefore, the generalization of the findings may not be precise as the study included a low number of cases collected in three months. Further study for an extended period including more populations from different groups is recommended to generate the baseline information to address the magnitude of the disease.

## Conclusions

From this study, it can be concluded that HMPV is an important human pathogen associated with ARIs in young children. HMPV can cause a wide range of respiratory tract illnesses ranging from the minor upper respiratory tract to severe bronchiolitis including pneumonia and asthma among children under the age of 15 years. The severity is higher in young children as compared to older children often leading to hospitalization. Likewise, HMPV co-infection with RSV and Parainfluenza virus was also reported in our study indicating HMPV infection as one of the predominant causes of ARIs in children in Kathmandu. The study finally suggests immediate care in case of the flu-like syndrome among children to avoid the potential respiratory severity of HMPV.

## Data Availability

The raw data of the study will be available on request to the corresponding author at upendrats@gmail.com/upendra.thapashrestha@cdmi.tu.edu.np.

## Abbreviations

ARI: Acute Respiratory Tract Infection
CDC: Center for Disease Control and Prevention
cDNA: Complimentary DNA
COPD: Chronic Obstructive Pulmonary Disease
Ct: Cycle threshold value
DNA: Deoxyribonucleic acid
HMPV: Human Metapneumovirus
NC: Negative control
PC: Positive Control
RTIs: Respiratory Tract Infections
RNA: Ribonucleic acid
RSV: Respiratory Syncytial Virus
RT-PCR: Reverse Transcriptase Polymerase Chain Reaction
URTIs: Upper Respiratory Tract Infections
VTM: Viral Transportation Media
